# Genome sequence of a human monkeypox virus isolate from Central Europe during the 2022 outbreak

**DOI:** 10.1128/mra.00415-25

**Published:** 2025-10-21

**Authors:** Ármin Gergely Nagy, Ágota Ábrahám, István Prazsák, Balázs Kakuk, Brigitta Zana, Ágnes Nagy, Dóra Tombácz, Gábor Kemenesi, Zsolt Boldogkői

**Affiliations:** 1 Department of Medical Biology, University of Szeged, Albert Szent-Györgyi Medical School 37442, Szeged, Hungary; 2 National Laboratory of Virology, Szentágothai Research Centre, University of Pécs 37656, Pécs, Hungary; 3 Biological Laboratory of the Hungarian Defense Forces, Budapest, Hungary; 4 Faculty of Sciences, University of Pécs, Institute of Biology 37656, Pécs, Baranya, Hungary; Katholieke Universiteit Leuven, Leuven, Belgium

**Keywords:** monkeypox virus, genome sequencing, nanopore sequencing, long-read sequencing, amplicon sequencing

## Abstract

We report the genome sequence of a human monkeypox virus isolated from an early Central European case. The 197,993 bp genome was assembled from amplicons sequenced using the Oxford Nanopore method. It has a GC content of 32.9% and belongs to subclade IIb B.1.3.

## ANNOUNCEMENT

The human monkeypox virus (hMPXV) is a zoonotic virus in the *Orthopoxvirus* genus of the *Poxviridae* family (1, 2), closely related to the smallpox-causing variola virus (3). In August 2024, the World Health Organization declared hMPXV a public health emergency of international concern, and it is now listed among emerging pathogens with pandemic potential (4, 5).

To support a better understanding of hMPXV evolution, we sequenced the virus isolate MPXV_NRL_4279/2022, obtained in 2022, from a patient’s skin lesion in Prague, Czechia.

The virus was propagated under BSL-4 conditions at the National Laboratory of Virology, University of Pécs, and passaged once in Vero cells (ATCC, CCL-81) to produce sufficient quantity of infectious material. DNA extraction was carried out using the Direct-zol RNA MiniPrep kit (Zymo Research, USA), following the manufacturer’s instructions except for DNase I treatment.

A set of 22 multiplex PCR primer pairs was designed to generate ~10 kb overlapping amplicons covering the viral genome. Primer sequences along with the detailed protocol are available on protocols.io (6). Briefly, repliQa HiFi ToughMix (Quantabio) was used to amplify complex and repetitive genomic regions from fragments with 50–100 bp overlaps. Two distinct PCR rounds were performed, each targeting non-overlapping segments. PCR products were purified using SPRI beads (AmpureXP) following the manufacturer’s instructions. Sequencing libraries were prepared with the SQK-LSK110 kit (Oxford Nanopore Technologies, ONT) and barcoded using the EXP-NBD196 kit. Sequencing was performed on a MinION Mk1B device with an R9.4.1 flow cell (FLO-MIN106), yielding 29,404 reads with an N50 of 9,556 (Table 1).

**TABLE 1:** Sequencing summary and structural variations of MPXV_NRL 4279/2022 isolate^*a*^^,*b*^

Metrics type	Metrics value	Variant type	Position	Length (bp)	Sequence	Affected gene/Location
Number of Reads	29 404	Substitutions	36850–36851	2	AT →TA	OPG055
			64426	1	C → T	IGR
Mean Depth	1309,92		111074	1	C → T	OPG130
			190660	1	G → A	OPG016
Mean BaseQ	23,8	Deletions	607–609	3	TTT	IGR
			133177–133186	10	CAATCTTTCT	IGR
Mean MapQ	60		150586–150602	17	GATATGATGGATATGAT	IGR
			196615–196617	3	AAA	IGR
N50	9556	Insertions	0	118	#^*c*^	ITR
			133102	1	T	CGR
Min. Amplicon Size	5004 bp		173276	5	ATATA	CGR
			197209	113	#	ITR
Max. Amplicon Size	30 665 bp	VNTR Insertions	4806	128	[TAACTAACTTATGACT]*8	ITR
			179243	324	[CATTATATA]*36	CGR
			192530	128	[AAGTCATAAGTTAGTT]*8	ITR

^*a*^ Summary of amplicon sequencing metrics and substitutions, deletions as well as insertions in theconsensus sequence compared to the NC_063383NC_063383NC_063383, reference genome.

^*b*^ bp, base pair; BaseQ, basecalling quality score; CGR, core genomic region; IGR, intergenic region; ITR, inverted terminal repeat; MapQ, mapping quality; VNTR, variable number of tandem repeat.

^*c*^ “#” indicates TTTTTTCTAGACACTAAATAAAATAGTAAAATATAATATTAATGTACTAAAACTTATGTATTATTAATTTATCTAACTAAAGTTAGTAAATTATATACATAA.

Basecalling using the super-accurate model and adapter trimming was performed with Guppy 6.5.7 (7), and low-quality reads were filtered out using NanoFilt2.8.0. *De novo* assembly using Raven 1.8.3 (8) produced two contigs, which were merged into a single sequence using RagTag 2.1.0 (9).

To determine the exact genomic termini, the results of an additional direct RNA-sequencing of our isolate (10) were utilized to generate a consensus sequence using samtools (11). The consensus matched the termini of the closely related, highly accurate OXO44336.2 genome (12).

The complete, assembled genome is 197,993 bp in length and has a 32.9% GC content. It shows no frameshift mutations or premature stop codons, indicating no major disruptions in protein-coding regions. Comparison with the reference genome (NC_063383) revealed four deletions totaling 33 bp, all located in intergenic regions. Five nucleotide substitutions were identified, three of which resulted in amino acid changes within the OPG055 (F11), OPG130 (A5L), and OPG016 (N3R) genes (Table 1). Additionally, seven insertions were detected: two at the terminal ends, three within variable number tandem repeat regions (VNTRs), and two small insertions (Table 1). Whole-genome phylogenetic analysis using Nextclade v3.10.2 (13) indicated that the MPXV_NRL_4279/2022 isolate belongs to Clade IIb B.1.3 of hMPXV. This genome, associated with the 2022 multi-country outbreak, provides valuable insight into the evolutionary dynamics of poxviruses.

## Data Availability

The sequence has been deposited in the NCBI Viral Genomes database under the GenBank accession number PV424067. Raw sequencing reads are available under BioProject ID PRJNA1241293 and have been deposited in the Sequence Read Archive (SRA) under accession number SRP572719.
